# *Pseudomonas syringae* pv. *actinidiae* from Recent Outbreaks of Kiwifruit Bacterial Canker Belong to Different Clones That Originated in China

**DOI:** 10.1371/journal.pone.0057464

**Published:** 2013-02-27

**Authors:** Margi I. Butler, Peter A. Stockwell, Michael A. Black, Robert C. Day, Iain L. Lamont, Russell T. M. Poulter

**Affiliations:** Department of Biochemistry, University of Otago, Dunedin, New Zealand; Institut Pasteur, France

## Abstract

A recently emerged plant disease, bacterial canker of kiwifruit (*Actinidia deliciosa* and *A. chinensis*), is caused by *Pseudomonas syringae pv. actinidiae* (PSA). The disease was first reported in China and Japan in the 1980s. A severe outbreak of PSA began in Italy in 2008 and has spread to other European countries. PSA was found in both New Zealand and Chile in 2010. To study the evolution of the pathogen and analyse the transmission of PSA between countries, genomes of strains from China and Japan (where the genus *Actinidia* is endemic), Italy, New Zealand and Chile were sequenced. The genomes of PSA strains are very similar. However, all strains from New Zealand share several single nucleotide polymorphisms (SNPs) that distinguish them from all other PSA strains. Similarly, all the PSA strains from the 2008 Italian outbreak form a distinct clonal group and those from Chile form a third group. In addition to the rare SNPs present in the core genomes, there is abundant genetic diversity in a genomic island that is part of the accessory genome. The island from several Chinese strains is almost identical to the island present in the New Zealand strains. The island from a different Chinese strain is identical to the island present in the strains from the recent Italian outbreak. The Chilean strains of PSA carry a third variant of this island. These genomic islands are integrative conjugative elements (ICEs). Sequencing of these ICEs provides evidence of three recent horizontal transmissions of ICE from other strains of *Pseudomonas syringae* to PSA. The analyses of the core genome SNPs and the ICEs, combined with disease history, all support the hypothesis of an independent Chinese origin for both the Italian and the New Zealand outbreaks and suggest the Chilean strains also originate from China.

## Introduction

*Pseudomonas syringae* pv. *actinidiae* (PSA) was first isolated and described in Japan in 1989 as the causal agent of bacterial canker of kiwifruit (*Actinidia deliciosa*) [1]. The disease manifests as brown leaf spots with chlorotic haloes, brown discoloration of buds, cankers with exudates on trunks and twigs, collapsed leaders and eventually the death of the plant [2]. A similar disease was described in 1989 in Sichuan Province of China [3] and subsequently in the Chinese provinces of Anhui, and Shaanxi (references cited in [4]). The disease was later found in Korea [5]. The first report from Europe was in Italy in 1992 [6]; this was followed by a more severe outbreak of bacterial canker in 2008 [7]. By 2010 the disease had spread to Portugal and France on both the green kiwifruit *A. deliciosa* and the gold kiwifruit *A. chinensis* [8], . In October 2010 (spring), bacterial canker of kiwifruit was found in Te Puke in New Zealand [10]. The outbreak has since spread from the Te Puke region (www.kvh.org.nz), although the disease is, so far, restricted to the North Island. PSA has also been found in Chile, in late 2010 [11].

The species complex *Pseudomonas syringae* has been divided on the basis of genome similarity into nine genomospecies [12] or five phylogroups [13]. Based on their host range and the type of symptoms they cause, the *Pseudomonas syringae* strains that are plant pathogens can be assigned to different pathovars. Although a group of strains from the same host may be assigned to the same pathovar type, the pathovar designation may not correspond to a natural phylogenetic group [13], [14]. For example, some of the strains that have been grouped together as *P. syringae* pv. *tomato* are genetically quite distinct (belonging to separate clades), even though they share a common host, the tomato plant [15]. *P. syringae* strains that are isolated from plants, but which do not cause disease, are not given a pathovar designation. For example, the non-pathogenic *P. syringae* Cit7 (Ps Cit7) was isolated from citrus fruit trees. There has been considerable variation in the nomenclature used to describe different strains of PSA [16]. In the present study the informal name PSA will be used and will be restricted to those *Pseudomonas syringae* pv. *actinidiae* that cause bacterial canker. PSA from the late Italian outbreak, the Chilean, the New Zealand and the Chinese strains form a very closely related group that has been described as belonging to a single genetic lineage [16]. In New Zealand, strains of *P. syringae* have been isolated from kiwifruit plants that showed no evidence of bacterial canker. These apparently non-pathogenic strains are genetically quite distinct from PSA [16], and will be referred to in this report by the informal names PsD (for *Ps deliciosa*) and PsHa (for *Ps* “Hayward”, the most common cultivar of *A. deliciosa*) without a pathovar designation. PsD has also been isolated in Australia [17]. The strains referred to here as PsD have previously been referred to as PSA-LV [16] and the “Asian” PSA [10], [17], although no isolates have been reported from Asia.

Comparative genomics of strains from the *P. syringae* species complex indicates that their genomes have a shared set of genes (the core genome) common to all, or almost all, strains. Interspersed within the core genomes of these strains are blocks of DNA (the ‘accessory genome’) that occur in some strains but not others [15]. The accessory genomes of strains from the *P. syringae* species complex have been shown to include genomic islands and various mobile elements such as insertion sequences (IS elements) [18], [19], transposons and integrative conjugative elements (ICEs) [19], [20]. ICEs are self-transmissible mobile genetic elements integrated into the genomes of some bacteria. They encode the machinery for conjugation as well as regulatory systems to control their excision from the chromosome, their conjugative transfer and their integration [21]. The sequences flanking ICEs are often referred to as the island attachment sites (*attL* and *attR*) [21]. The presence of the flanking *att* sites reflects the integration of an ICE into its target site, often a tRNA gene (*attL* is a full length tRNA gene, while *attR* is a truncated version). Excision of an ICE yields a covalently closed circular molecule that can be transferred to a new host by the conjugation machinery. ICEs can participate in horizontal transfer between related strains of bacteria. They often contribute to the phenotype of their host by altering the host range or conferring resistance or adaptation to some environmental factor [22]. Mazzaglia et al., [23] described sequences in strains of PSA that were similar to PPHGI-1, a genomic island found in *P. syringae* pv. *phaseolicola* strain 1302A. Although Mazzaglia et al., [23] recognised that the PSA sequences probably represented an island, they did not describe the limits of the islands or establish that they were ICEs.

In phylogenetic and epidemiological analyses, such as the determination of the source of an emerging pathogen or the routes of disease transmission, it is necessary to have a method of distinguishing strains from one another. Studies using multi-locus sequence typing (MLST), various methods of molecular fingerprinting and genome sequencing have revealed that PSA isolates from Japan and Korea are two distinct genetic lineages, while strains from China, Italy (the recent, severe outbreak), Chile and New Zealand represent a third, very closely related group of strains [16], [23], [24]. The hypothesis that PSA may have originated and co-evolved with the *Actinidia* genus in China is supported by recent genome sequence data [23]. To analyse the geographic origin of PSA we completely sequenced and compared the genomes of PSA strains from Japan, Chile, China, Italy and New Zealand. We have supplemented this whole genome data with amplicon sequencing of multiple loci in additional strains. We have also included in the analysis data from whole genome sequencing of PSA present in the public databases. The analysis of the SNPs present in the core genomes and accessory genomes enable us to separate these PSA strains into distinct geographical groups. Our results also indicate that China is the source of the present outbreaks.

## Results

### Strains and Genomes

In the present study, we were especially interested in analysing the genomes of PSA strains from the outbreak in New Zealand, and if possible, tracing the origin of this outbreak. We sequenced the genomes of four New Zealand PSA strains involved in serious damage to kiwifruit vines, two strains from Shaanxi province in China isolated in 2010 (M7 and M228), one strain isolated in Italy in 2010, two strains from Chile (isolated in 2011) and a strain from Japan, isolated in 1984 (Table 1). Three *Pseudomonas syringae* (two PsD strains and one strain of PsHa), isolated from kiwifruit, but not associated with bacterial canker were also fully sequenced. Genome coverage and genome assembly data for each genome are summarized in Table 2. The whole genome sequencing was supplemented by amplicon sequencing of various loci from a further 10 PSA strains from New Zealand, three strains from Chile and three from Italy. In addition, amplicon sequencing was applied to selected loci from an additional four PsD and three PsHa isolates.

**Table 1:** PSA and other *P. syringae* strains used in this study for genome sequencing or for analysis with genome-derived SNP markers.

Isolate	Alternative name	Region of Origin	Country	Isolation Date	Strain Source	Genome Data	Amplicon sequencing
**PSA**							
ICMP 18800		Paengaroa	NZ	Nov 2010	LandCare, NZ	NZGL	
ICMP 18708		Te Puke	NZ	Nov 2010	LandCare, NZ	NZGL	
TP1		Te Puke	NZ	July 2011	IIL, RTMP	NZGL	
TP2		Te Puke	NZ	July 2011	IIL, RTMP		Otago GAS
3.1		Te Puke	NZ	July 2011	IIL, RTMP		Otago GAS
2.2		Te Puke	NZ	July 2011	IIL, RTMP		Otago GAS
6.1		Te Puke	NZ	July 2011	IIL, RTMP	NZGL	
AC163		Te Kaha	NZ	Feb 2012	VLS, Te Puke		Otago GAS
AC164		Te Kaha	NZ	Feb 2012	VLS, Te Puke		Otago GAS
AC165		Te Kaha	NZ	Feb 2012	VLS, Te Puke		Otago GAS
AC166		Te Kaha	NZ	Feb 2012	VLS, Te Puke		Otago GAS
AC507		Oropi	NZ	Feb 2012	VLS, Te Puke		Otago GAS
ICMP 18839		Bay of Plenty	NZ	Feb 2011	LandCare, NZ		Otago GAS
ICMP 18875		Bay of Plenty	NZ	Feb 2011	LandCare, NZ		Otago GAS
ICMP 18743	CRA-FRU11.40	Rome	Italy	2010	LandCare, NZ		Otago GAS
ICMP 18744	CRA-FRU11.41	Rome	Italy	2010	LandCare, NZ	NZGL	
ICMP 18745	CRA-FRU11.45	Latina	Italy	2010	LandCare, NZ		Otago GAS
ICMP 18746	CRA-FRU11.43	Latina	Italy	2010	LandCare, NZ		Otago GAS
ICMP 19438		VII Region of Maule	Chile	2010	LandCare, NZ		Otago GAS
ICMP 19439		VII Region of Maule	Chile	2010	LandCare, NZ	NZGL	
ICMP 19455		VII Region of Maule	Chile	2010	LandCare, NZ	NZGL	
ICMP 19456		VII Region of Maule	Chile	2010	LandCare, NZ		Otago GAS
ICMP 19457		VII Region of Maule	Chile	2010	LandCare, NZ		Otago GAS
M7	CH2010-6	Wei County, Shaanxi	China	Jun 2010	ZZ, LH	NZGL	
M228		Wei County, Shaanxi	China	Dec 2010	ZZ, LH	NZGL	
ICMP 9853	KW1	Shizuoka	Japan	May 1984	LandCare, NZ	NZGL	
M302091	FTRS L1	Kanagawa	Japan	1984	AEAL	[25]	
CRA-FRU8.43	I-2	Latina	Italy	2008	AFTG	[2]	
NCCP3871	I-1	Roma province	Italy	1992	AFTF	[2]	
NCCP3739	KW11	Shizuoka	Japan	1984	AFTH	[2]	
PA459			Japan	1988	AGNQ	[23]	
KW41	ICMP 9855	Shizuoka	Japan	1984	AGNP	[23]	
CFPB7286	PSAH108	Lazio, Latina	Italy	2008	AGNO	[23]	
CH2010-6		Shaanxi	China	2010	AGUH	[23]	
**PsD**							
ICMP 18804	PsD	Te Puke	NZ	Nov 2010	LandCare, NZ	NZGL	
ICMP 18806	PsD	Pongakawa	NZ	Nov 2010	LandCare, NZ	NZGL	
ICMP 18802	PsD	Paengaroa	NZ	Nov 2010	LandCare, NZ		Otago GAS
ICMP 18803	PsD	Hawke’s Bay	NZ	Nov 2010	LandCare, NZ		Otago GAS
ICMP 18882	PsD	Motueka	NZ	Nov 2010	LandCare, NZ		Otago GAS
ICMP 18883	PsD	Anatoki, Golden Bay	NZ	Nov 2010	LandCare, NZ		Otago GAS
**PsHa**							
ICMP 18807	PsHa1	Tauranga	NZ	Nov 2010	LandCare, NZ	NZGL	
	PsHa2	Bay of Plenty		May 2012	VLS, Te Puke		Otago GAS
	PsHa3	Bay of Plenty		May 2012	VLS, Te Puke		Otago GAS
	PsHa4	Bay of Plenty		May 2012	VLS, Te Puke		Otago GAS

Strain sources are Landcare Research, NZ, http://www.landcareresearch.co.nz/resources/collections/icmp; Verified Laboratory Services (VLS) at Te Puke, http://www.vls.net.nz; or held by the authors. Zhibo Zhao and Lili Huang at Northwest A&F University, China, supplied genomic DNA.

**Table 2:** Genome sequencing and assembly data for the ten fully sequenced *Pseudomonas syringae* pv. *actinidiae* (PSA) strains, two PsD strains and one PsHa.

Isolate	Genbank Accession^1^	Number ofcontigs	N50 (nt)	Longest contig(nt)	Total length(nt)	Depth of genomecoverage
ICMP9853	ANJB00000000	442	26,957	121,081	6,094,387	>300x
M7	ANJJ00000000	1,590	4,561	57,016	6,006,626	>30x
M228^2^	ANJI00000000	3,259	1,278	15,803	5,291,125	>100x
ICMP 18744	ANGD00000000	442	35,308	143,214	6,283,916	>300x
ICMP 18800	ANJD00000000	463	30,938	88,251	6,277,085	>300x
ICMP 18708	ANJC00000000	445	32,941	139,630	6,275,908	>300x
TP1	ANJG00000000	472	30,598	138,717	6,288,252	>300x
6.1	ANJH00000000	483	31,049	143,214	6,281,627	>300x
ICMP 19439	ANJM00000000	477	36,822	142,020	6,232,041	>50x
ICMP 19455	ANJK00000000	415	39,400	127,854	6,231,941	>50x
ICMP 18804^3^	ANJE00000000	263	52,055	224,101	6,261,251	>300x
ICMP 18806^3^	ANJF00000000	284	41,975	134,790	6,266,634	>300x
ICMP 18807^4^	ANJL00000000	148	90,428	274,756	6,129,794	>300x

1 These Whole Genome Shotgun projects have been deposited at DDBJ/EMBL/GenBank under the accessions listed above. The versions of the assemblies described in this paper are the first versions.

2 The sequence of this strain (M228) was generated from a limited quantity of genomic DNA. Although the genome coverage is adequate (>100x), the assembly is more fragmented and the exclusion of small contigs results in an artefactually low total length.

3 *P. syringae* PsD.

4 *P. syringae* PsHa.

Comparative genomic analyses were also applied to a draft genome sequence of the PSA pathotype strain M302091 [25] isolated from *A. deliciosa* in Japan in 1984 and draft genome sequences of Italian PSA strains, one isolated in 1992 (NCPPB 3871) and one isolated in 2008 (NCPPB 3739) [2]. Five draft genomes of PSA were available from two additional strains isolated in Japan (KW41, PA459), from strain CH2010-6 isolated from the province of Shaanxi in China (this strain is the same isolate as M7, L. Huang, pers. comm.) and one strain isolated in Italy (CFPB7286 in 2008) [23].

### Phylogenetic Relationship of PSA with Other *P. syringae*

**Figure 1:**
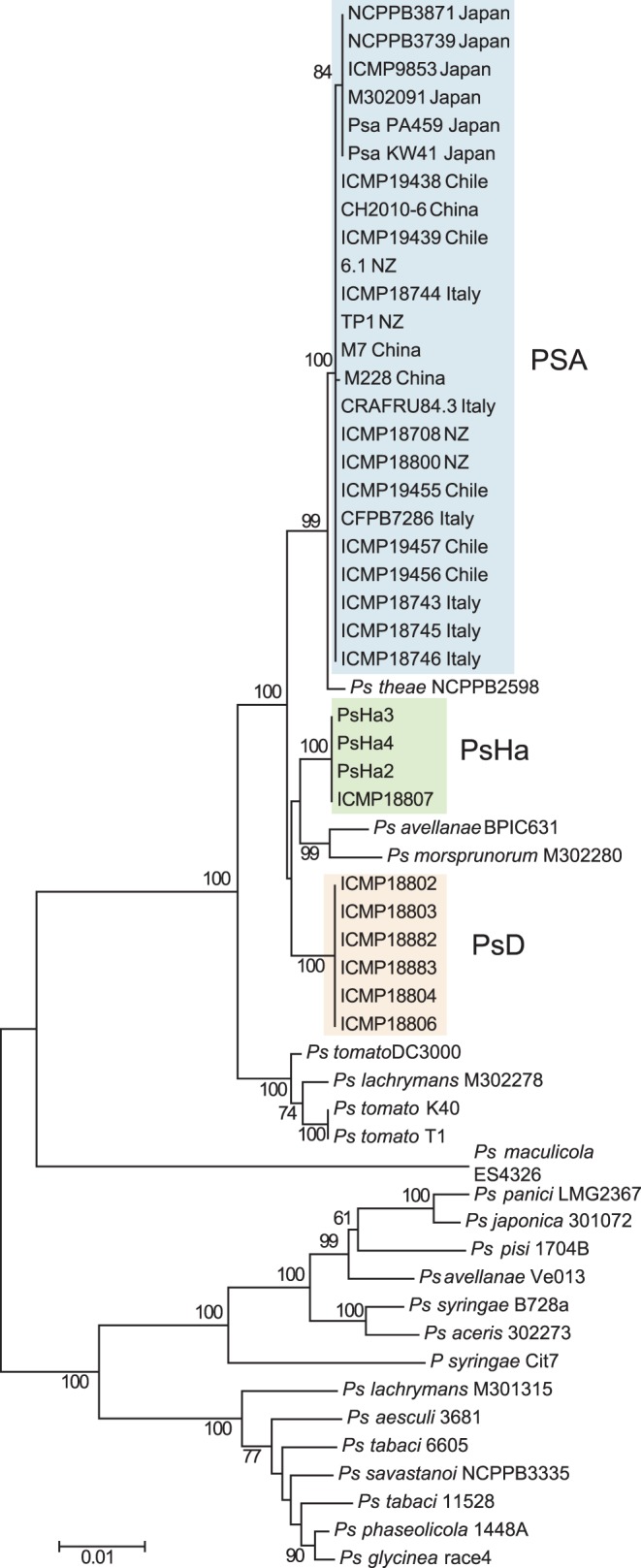
Evolutionary relationships of *P. syringae* pv. *actinidiae* and members of the *P. syringae* species complex. Phylogenetic tree constructed using concatenated sequences of four core genome loci (gapdh, gltA, gyrB and rpoD), with bootstrap values greater than 75 per cent (1050 replicates) shown at the nodes. The evolutionary history was inferred using the Neighbor-Joining method in MEGA5 [26]. The PSA strains are enclosed by a blue shaded box, with their strain number and country of origin shown. PsD strains are enclosed by an orange shaded box and PsHa strains are enclosed by a green shaded box. The strain numbers of other members of the *P. syringae* complex are also shown.

Sequences from four loci of the core genomes (rpoD, sigma factor 70; gyrB, DNA gyrase B; gltA citrate synthase; and gapA, glyceraldehyde-3-phosphate dehydrogenase) of PSA and other strains within the *P. syringae* species complex were concatenated and aligned. The alignment was used to construct a phylogenetic tree (Figure 1). All of the PSA strains are clustered together on this phylogenetic tree. The Japanese PSA strains are distinct from the group containing PSA strains from the recent, severe Italian outbreak, the New Zealand, Chile and the Chinese strains. This analysis agrees with the results of Chapman et al., [16]. *Pseudomonas syringae* pv. *theae* is the most closely related strain to PSA. The PSA/*P. syringae* pv. *theae* group has three sister clades, one comprising *P. avellanae* BP631 and *P. syringae* pv. *morsprunorum*, a second consisting of the PsD strains and a third group composed of the PsHa strains. The phylogenetic tree, therefore, confirms that the PsD and PsHa strains are only distantly related to the virulent PSA implicated in the recent outbreaks of bacterial canker in kiwifruit.

### New Zealand, Chilean, Italian (Recent Outbreak) and Chinese PSA Strains are Very Similar but not Identical to Each Other

Since the comparison of the four loci of the core genome could not distinguish among the New Zealand, Chilean, Italian (recent outbreak) and Chinese PSA strains, we performed whole genome sequencing of selected PSA strains to attempt to determine their phylogenetic relationships. The sequence of these strains was compared, together with sequences from PSA strains present in Genbank.

SeqMonk representations of the whole genome sequences aligned with the fully assembled and annotated genome of *Pseudomonas syringae* pv. *tomato* DC3000 were generated (examples shown in Figure S1 with a subset expanded in Figure S2). SeqMonk is a program that enables the visualisation and analysis of mapped next generation sequence data. Of the fully assembled *P. syringae* genomes, that of *Pseudomonas syringae* pv. *tomato* DC3000 is the most similar to PSA and was used as a reference for the SeqMonk comparisons. The SeqMonk displays demonstrate that the PSA strains are very similar to each other, although the Japanese PSA is clearly different from the other PSA strains.

Single nucleotide polymorphisms (SNPs) were identified using Blast2 alignments. To obtain reliable SNPs, we used only those contigs greater than 10 kb. The presence of large numbers of repeated sequences is the main difficulty in assembling the PSA genome. The contigs frequently end with repetitious sequences; consequently, the ends of the contigs are prone to mis-assembly. For this reason we did not include the first and last 60 bp of each contig in this analysis. We also compared the SNP-containing loci with the corresponding loci in the Japanese PSA strains. This analysis was integrated with sequences available in Genbank and additional amplicon sequencing of other PSA.

Five SNPs were specific to all 14 New Zealand isolates, another set of three SNPs were specific to all six Italian strains (one of the Italian-specific SNPs has previously been described by Mazzaglia et al., [23]), while ten SNPs were specific to the five Chilean strains (Tables 3, 4 and Table S1). These three sets of SNPs from strains sourced to distinct geographical areas (Italy, New Zealand and Chile) were not present in either of the Chinese strains we analysed, nor in the Japanese strain. These three geographically separated clones are, however, very closely related since they differ by approximately one base per Mb. The Chinese strains (M7/CH2010-6, CH2010-5 and CH2010-7) are also very closely related to the geographical clones, with a similar frequency of SNPs. In contrast, the strain M228 is more distantly related to the geographical clones, differing at approximately one SNP per 16 kb.The sequences of the SNPs characteristic of each geographical clone were confirmed in all strains by amplicon sequencing.

**Table 3:** Single nucleotide polymorphisms (SNPs) distinctive of New Zealand and Italian PSA strains.

		M7 contigs						
		6	40	12	492	192	290	195
CH2010-6^1^	Shaanxi	T	C	T	C	G	insertion	A
M7^1^	Shaanxi	T	C	T	C	G	insertion	A
M228	Shaanxi	T	C (*1)	T	C	G	insertion	A
ICMP18800	Paengaroa, NZ	T	C	A	T	A	22 bp del	G
ICMP18708	Te Puke, NZ	T	C	A	T	A	22 bp del	G
TP1	Te Puke, NZ	T	C	A	T	A	22 bp del	G
TP2	Te Puke, NZ	T	C	A	T	A	22 bp del	G
3-1	Te Puke, NZ	T	C	A	T	A	22 bp del	G
2-2	Te Puke, NZ	T	C	A	T	A	22 bp del	G
6-1	Te Puke, NZ	T	C	A	T	A	22 bp del	G
AC163	Te Kaha, NZ	T	C	A	T	A	22 bp del	G
AC164	Te Kaha, NZ	T	C	A	T	A	22 bp del	G
AC165	Te Kaha, NZ	T	C	A	T	A	22 bp del	G
AC166	Te Kaha, NZ	T	C	A	T	A	22 bp del	G
AC507	Oropi, NZ	T	C	A	T	A	22 bp del	G
ICMP18839	Te Puke, NZ	T	C	A	T	A	22 bp del	G
ICMP18875	Te Puke, NZ	T	C	A	T	A	22 bp del	G
ICMP18743	Italy	C	T	T	C	G	insertion	A
ICMP18744	Italy	C	T	T	C	G	insertion	A
ICMP18745	Italy	C	T	T	C	G	insertion	A
ICMP18746	Italy	C	T	T	C	G	insertion	A
CRAFRU8.43	Italy	C	T	T	C	G	insertion	A
CFBP7286	Italy	C	T	T	C	G	insertion	A
ICMP19438	Chile	T	C	T	C	G	insertion	A
ICMP19439	Chile	T	C	T	C	G	insertion	A
ICMP19455	Chile	T	C	T	C	G	insertion	A
ICMP19456	Chile	T	C	T	C	G	insertion	A
ICMP19456	Chile	T	C	T	C	G	insertion	A
NCCP3871	Italy (1992)	T	C (*2)	T (*1)	C (*2)	G	insertion	A
NCCP3739	Japan	T	C (*2)	T (*1)	C (*2)	G	insertion	A
M302091	Japan	T	C (*2)	T (*1)	C (*3)	G	insertion	A
ICMP9853	Japan	T	C (*2)	T (*1)	C (*2)	G	insertion	A
KW41	Japan	T	C (*2)	T (*1)	C (*2)	G	insertion	A
PA459	Japan	T	C (*2)	T (*1)	C (*2)	G	insertion	A
Ps theae NCPPB2598		T	C (* 8)	T (*6)	C	G (*1)	insertion	A (*4)

1 M7 and CH2010-6 are the same isolate, sequenced independently.

* indicates a further SNP is present in this locus.

**Table 4:** Single nucleotide polymorphisms (SNPs) distinctive of PSA strains from Chile.

		ICMP19439 contigs including the SNP position in contigs with multiple SNPs									
		1	3∶25 Kb^1^	3∶29 Kb	3∶30 Kb	9∶45 Kb	9∶104 Kb	90	92	96	102
Strain	Origin										
ICMP19438	Chile	T	cttctag x 8	A	A	A	A	A	T	T	A
ICMP19439	Chile	T	cttctag x 8	A	A	A	A	A	T	T	A
ICMP19455	Chile	T	cttctag x 8	A	A	A	A	A	T	T	A
ICMP19456	Chile	T	cttctag x 8	A	A	A	A	A	T	T	A
ICMP19457	Chile	T	cttctag x 8	A	A	A	A	A	T	T	A
CH2010-6	Shaanxi	C	cttctag x 9	G	C	G	T	C	G	G	C
M7	Shaanxi	C	cttctag x 9	G	C	G	T	C	G	G	C
M228	Shaanxi	C	cttctag x 6	G	C	G	T	C	G	G	C
6-1	NZ	C	cttctag x 9	G	C	G	T	C	G	G	C
ICMP18800	NZ	C	cttctag x 9	G	C	G	T	C	G	G	C
ICMP18708	NZ	C	cttctag x 9	G	C	G	T	C	G	G	C
TP1	NZ	C	cttctag x 9	G	C	G	T	C	G	G	C
ICMP18744	Italy	C	cttctag x 9	G	C	G	T	C	G	G	C
CRAFRU8.43	Italy	C	cttctag x 9	G	C	G	T	C	G	G	C
CFPB7286	Italy	C	cttctag x 6	G	C	G	T	C	G	G	C
NCCP3871	Italy	C	cttctag x 2	G	C	G	T	C	G	G	C
NCCP3739	Japan	C	cttctag x 2	G	C	G	T	C	G	G	C
M302091	Japan	C	cttctag x 2	G	C	G	T	C	G	G	C
ICMP9853	Japan	C	cttctag x 2	G	C	G	T	C	G	G	C
PA459	Japan	C	cttctag x 3	G	C	G	T	C	G	G	C
*Ps theae*		C	absent	G	C	G	T	C	G	G	C

1 The polymorphism at ∼25 Kb on contig3 is not a simple SNP, it is a variable number microsatellite.

We included in our SNP analyses sequences amplified from the loci used by Mazzaglia et al. [23]. Our sequences are in complete agreement with the previous analysis except the SNP present on scaffold 190, contig 7. The Italian strains carried a C at this locus [23]. However, whereas Mazzaglia et al. [23] report that the New Zealand strains ICMP18839 and ICMP18875 also have a C at this site, we found that all of the New Zealand strains we have analysed, including ICMP18839 and ICMP18875, have a T at this locus (Table S1). The Italian strains therefore carry a distinctive SNP at this position. There were a small number of idiosyncratic SNPs unique to particular strains. While such SNPs are not phylogenetically informative, they do provide an indication of the rate of acquisition of *de novo* mutations in the genome. The idiosyncratic SNPs of the New Zealand strains, based on the comparison of about 5 Mb, are listed in Table S2. Comparison of ∼4.5 Mb of the two, fully sequenced strains from Chile shows five idiosyncratic SNPs; in every case the derived allele was carried by strain ICMP19439. These results indicate that the clonal populations in both New Zealand and Chile are undergoing divergence, but as yet the frequency of idiosyncratic SNPs is less than one per Mb. This is similar to the situation in the Italian clone [23]. As mentioned above, the two fully sequenced Chinese strains (M7 and M228) show greater divergence (about one SNP per 16 kb).

### Integrative Conjugative Elements (ICEs) in PSA

Mazzaglia et al., [23] noted that one region of the genomes of the PSA strains seemed highly divergent. They recognized this region of difference as resembling the genomic island PPHGI-1, which is an ICE, in *P. syringae* pv. *phaseolicola* strain 1302A [20]. The sequences flanking PPHGI-1 in *P. syringae* pv. *phaseolicola* are the attachment sites (*attL*, the full length lysine tRNA and *attR*, the truncated version), reflecting the integration of the island into a lysine tRNA gene [20]. PPHGI-1 island is broadly syntenic with PsyrGI-6, a genomic island in the fully assembled genome of *P. syringae* pv. *syringae* B728a [19]. This synteny is illustrated in Figure S3, with an Artemis Comparison Tool alignment (ACT) [27]. The *P. syringae* pv. *syringae* PsyrGI-6 sequence is flanked by sequences identical to the *P. syringae* pv *phaseoliola* PPHGI-1 lysine tRNA *attL* and *attR*. The ACT alignment indicates that PsyrGI-6 from *P. syringae* pv. *syringae* is broadly syntenic with PPHGI-1, however PsyrGI-6 shows closer resemblance to PSA sequences than PPHGI-1 and it was therefore used for comparative analyses with PSA. Related islands were detected in each of the recent Italian, New Zealand, Chilean and Chinese strains of PSA. The limits of the islands were defined by the flanking lysine tRNA gene and truncated lysine tRNA sequences. We could not find any sequences in the PsD strains or ICMP18807 (PsHa1) that were similar to PPHGI-1 or PsyrGI-6.

**Figure 2:**
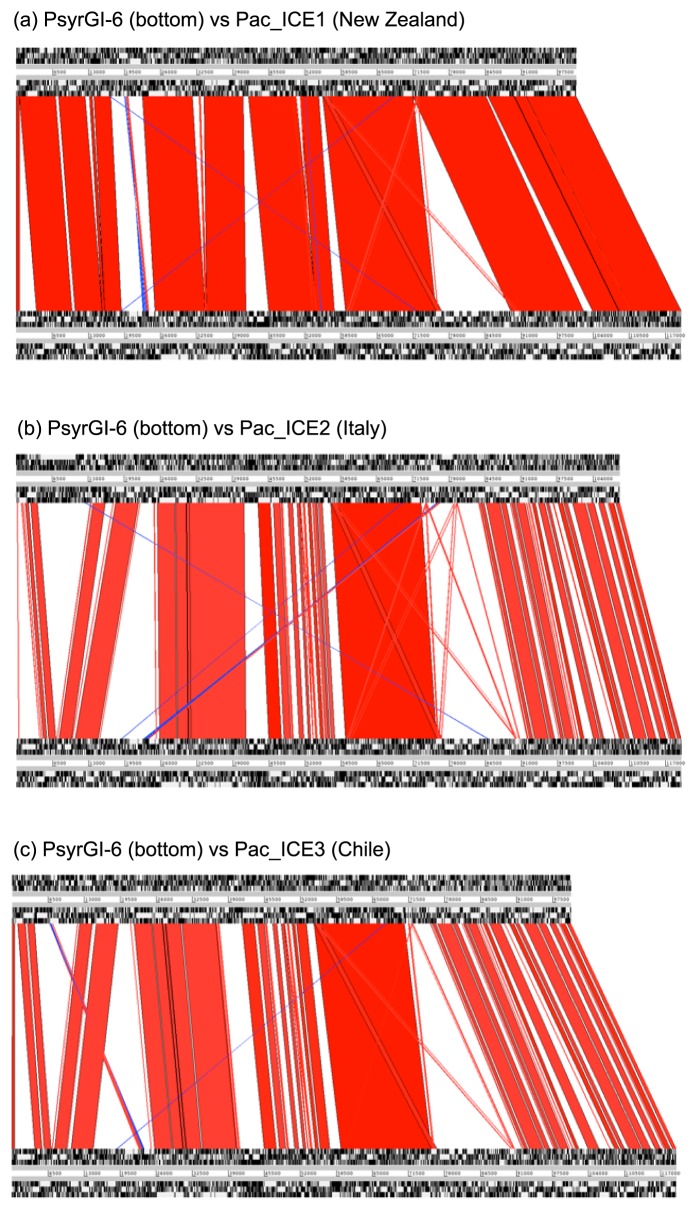
Comparison of the island in *P. syringae* pv. *syringae* B728a (PsyrGI-6) with those in PSA. Artemis Comparison Tool [27] alignments of PsyrGI-6 with (a) the island in ICMP18708 (NZ) (Pac_ICE1), (b) ICMP18744 (Italy)(Pac_ICE2) and (c) ICMP19455 (Chile)(Pac_ICE3). The blue areas refer to regions that are inverted between islands. Darker red regions are those that share the highest similarity.

Alignments of the island from *P. syringae* pv. *syringae* B728a with those from ICMP18708 (NZ), ICMP19455 (Chile) and ICMP18744 (Italy) are shown in Figure 2. These alignments, together with the presence of the *att* sites and the broad synteny between the PsyrGI-6 and PSA sequences, indicate that the PSA strains carry genomic islands closely similar to that of PsyrGI-6. Figure 2 also indicates that the island in ICMP18708 (NZ) is more similar to PsyrGI-6 than are the islands in the Italian (ICMP18744) or Chilean (ICMP19455) PSA strains.

**Figure 3:**
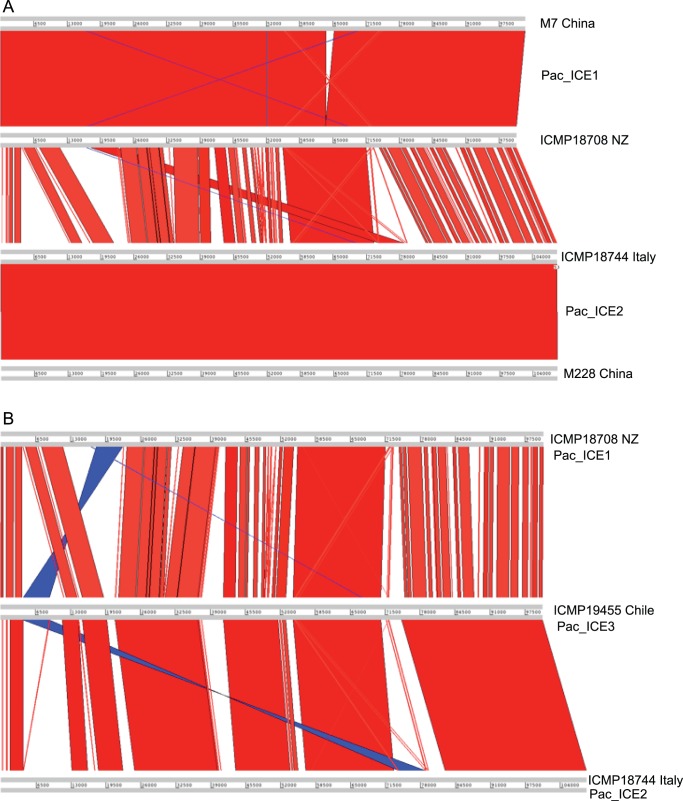
Comparative alignments of the islands in PSA. A. Comparison of Pac_ICEs from New Zealand and Italian PSA with Pac_ICEs from Chinese PSA strains. Upper panel: alignment of Pac_ICE1 from strain M7 (China) and ICMP18708 (New Zealand). Middle panel: alignment of Pac_ICE1 from ICMP18708 (NZ) and Pac_ICE2 from ICMP18744 (Italy). Lower panel: alignment of Pac_ICE2 from ICMP18744 (Italy) and M228 (China). B. Comparisons made with Pac_ICE3 from PSA from Chile. Upper panel: Comparison of Pac_ICE3 from ICMP19455 (Chile) with Pac_ICE1 from ICMP18708 (New Zealand). Lower panel: alignment of Pac_ICE3 from ICMP19455 (Chile) with that of Pac_ICE2 from ICMP18744 (Italy). The blue areas denote where two regions are inverted with respect to one another.

Artemis Comparison Tool alignments [27] between the islands in the PSA strains show that the islands in ICMP18708 (NZ), ICMP18744 (Italy) and ICMP19455 (Chile) are distinct, although they are broadly syntenic (Figure 3). The island in ICMP18708 (NZ) is almost identical to the island in strain M7 from China (Figure 3). The island sequence of ICMP18744 (Italy) is exactly the same as the sequence of the island in M228 (China) (Figure 3). Because these islands have numerous open reading frames predicted to be involved in integration and conjugation, and are flanked by attachment sites (*attL* and *attR*), we will therefore refer to them as integrative conjugative elements (ICEs). The ICEs found in *Pseudomonas syringae* pv. *actinidiae* will be referred to as Pac_ICE, following the system used to name the ICE in *Pseudomonas syringae* pv. *phaseolicola* PPHGI-1. The distinct ICEs will be referred to as Pac_ICE1 (from New Zealand strains and M7/CH2010-6, CH2020-5, CH2020-7 from China), Pac_ICE2 (from Italian strains and M228 from China) and Pac_ICE3 (in strains from Chile). The elements are ∼100 kb long, their annotated sequences have been submitted to Genbank and have the accession numbers shown in Table 5.

**Table 5:** Genbank accession numbers for examples of the integrative conjugative elements (ICEs) present in strains of PSA.

Pac_ICE name	PSA strain host	Country of PSA strain origin	GenBank Accession
Pac_ICE1_nz	ICMP18708	New Zealand	KC148184
Pac_ICE1_cn	M7 or CH2010-6	China	KC148185
Pac_ICE2_it	ICMP18744	Italy	KC148186
Pac_ICE2_cn	M228	China	KC148187
Pac_ICE3_cl	ICMP19455	Chile	KC148188

The annotations described in these accessions were provided by the RAST server (http://rast.nmpdr.org). Pac_ICE suffixes follow the internet domain name notation for country of origin of each PSA strain.

**Figure 4:**
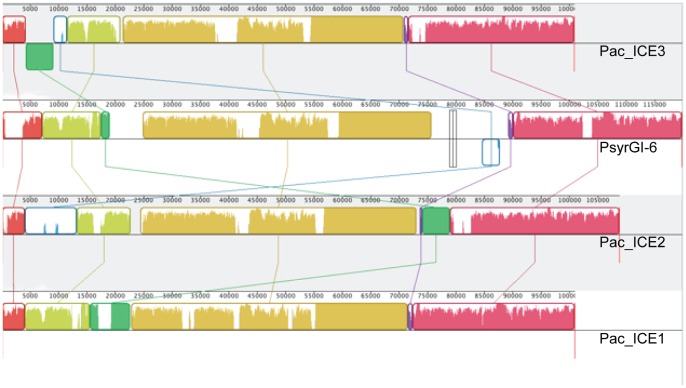
Mauve alignment of the genomic islands of PSA and *P. syringae* pv. *syringae* B728a. Depiction of Pac_ICE3 in ICMP19455 (Chile), PsyGI-6 in *P. syringae* pv. *syringae* B728a, Pac_ICE2 (ICMP18744, Italy) and Pac_ICE1 (ICMP18708, NZ) using Mauve [28]. Colored blocks each represent a locally co-linear block containing no apparent re-arrangements. Blocks below the centre line indicate regions that align in the reverse complement (inverse) orientation. Areas that are completely white were not aligned and probably contain sequence elements specific to a particular island. The height of the colour bars represents the average degree of sequence similarity.

**Figure 5:**
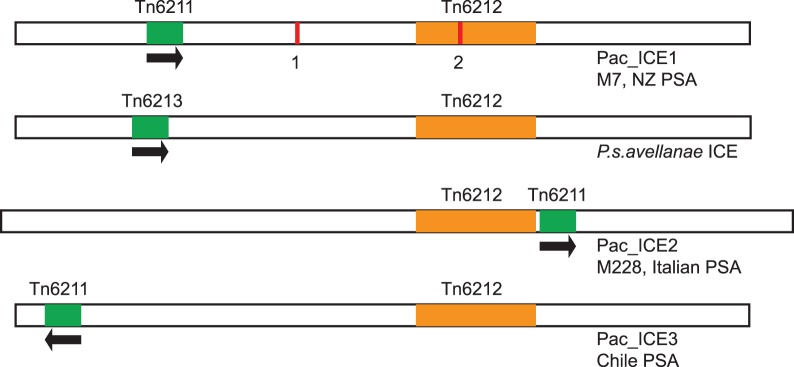
Depiction of mobile elements within the ICEs of PSA and *P. syringae* pv. *avellanae*. ICEs represented are Pac_ICE1 (top), present in PSA strains from New Zealand and in M7 (China); the ICE in *P. syringae* pv. *avellanae* Ve013; Pac_ICE2, present in PSA strains from Italy and in M228 (China) and Pac_ICE3, present in strains from Chile. Orange boxes indicate the ∼16 kb XerC transposon, Tn6212, present at almost 100% identity in the four ICEs shown and present in several ICEs in other *P. syringae* isolates/pathovars. Tn6211 is represented in PSA ICEs by a green box and an arrow showing the orientation of the three ORFs (including a methyl-accepting chemotaxis protein gene) present in these transposons. Tn6213 is an homologous element in *P. syringae* pv. *avellanae*, with ∼99% sequence identity to Tn6211. The red bars represent an IS element (1665 bp) which contains two overlapping ORFs with similarity to the transposases of IS3/IS911 family IS elements. It is present in only two of the ICEs examined so far, Pac_ICE1-nz in strain 6-1 at position1 and in Pac_ICE1-cn (in M7) at position2.

There are many examples of substantial insertions and deletions (indels) in the islands. For example, in Pac_ICE2 there is a 6 kb ORF not found in the Pac_ICE1 or Pac_ICE3. The sequences shared by the islands show variable similarity. A comparison of the ICE sequences using Mauve [28] (Figure 4) demonstrates that most of the sequences shared by the ICEs are significantly divergent (∼85% identical). However, there are two regions that are highly conserved. There is a transposon present at a different, characteristic position in the three different ICEs. The position of this transposon and its orientation are clearly demonstrated in the corresponding dot matrix alignments (Figure S4) and illustrated in Figure 5. This transposon sequence of 4875 bp is related to the IS sequence IS*Ppu19* of *Pseudomonas putida* and has been named Tn6211 at the Transposon Number Registry [29], [30]. Tn6211 carries, in addition to the IS*Ppu19*-related sequences, a methyl accepting chemotaxis protein gene. Although the Tn6211 sequences occupy distinct positions in each of the three Pac_ICE types, the sequences of the Tn6211 elements are more than 99% identical. The second conserved region (bases 55201–71516 in Pac_ICE1 from ICMP18708) has been designated Tn6212 [29] (Figure 5) and is almost identical in all of the Pac_ICEs. This 16317 bp transposon encodes a XerC-type tyrosine recombinase and includes multiple ORFs.

Comparison of the islands of the PSA strains reveals abundant phylogenetically informative differences between isolates from Italy, New Zealand, Chile and China. The islands of the different geographical isolates of PSA are very distinct. For example, comparison of the 73,600 base pairs that can be aligned between the islands in ICMP18708 (NZ) and ICMP18744 (Italy) reveals 10,203 SNPs (13.9% divergence). The divergence is not due to the sequences being non-functional and therefore subject to a high rate of substitution. Most of the sequence encodes uncorrupted predicted open reading frames. Comparison of seven of these ORFs in Pac_ICE1, Pac_ICE2 and Pac_ICE3 (Table S3) gave a dS/dN value much greater than one, suggesting that the ORFs are under strong, stabilizing selection (Table 6).

**Table 6:** dS/dN values gained from comparisons of seven ORFs in three Pac_ICEs.

		XerC/D		ICE_TraI		DSBA oxoreductase		uvrD helicase	
		ds/dn	ps/pn	ds/dn	ps/pn	ds/dn	ps/pn	ds/dn	ps/pn
Pac_ICE1	Pac_ICE2	NA	2.91	13.61	6.24	11.00	2.89	29.68	11.20
Pac_ICE1	Pac_ICE3	19.11	5.75	14.30	6.50	NA	3.17	29.68	11.20
Pac_ICE2	Pac_ICE3	NA	2.89	4.28	4.10	3.46	3.10	10.53	10.23
		**AdoMet methyl-transferase**		**DnaB**		**ParA**			
		**ds/dn**	**ps/pn**	**ds/dn**	**ps/pn**	**ds/dn**	**ps/pn**		
Pac_ICE1	Pac_ICE2	7.41	4.28	18.09	7.59	NA	8.40		
Pac_ICE1	Pac_ICE3	7.81	4.40	18.05	7.639	41.35	8.35		
Pac_ICE2	Pac_ICE3	6.23	6.17	12.91	12.70	1 substitution			
		**seven concatenated orfs**							
		**ds/dn**	**ps/pn**						
Pac_ICE1	Pac_ICE2	12.16	5.02						
Pac_ICE1	Pac_ICE3	14.82	6.08						
Pac_ICE2	Pac_ICE3	3.37	3.09						

The number of synonymous and non-synonymous substitutions were calculated using the SNAP website (www.hiv.lanl.gov). The name and position of the seven ORFs in Pac_ICE1 (ICMP18708, NZ), Pac_ICE2 (ICMP18744, Italy) and Pac_ICE3 (ICMP19455, Chile) that were used are shown in Table S3. dS/dN values were also calculated for codon aligned concatenations of the ORFs.

The comparison of Pac_ICE1 from the four, fully sequenced New Zealand strains (ICMP18708, ICMP18800, TP1 and 6.1) indicated that they are identical, except for the presence of an IS element of the type IS*3*/IS*911* in strain 6.1, beginning at position 40891 (Figure 5). This sequence has been designated IS*Psy31* at the IS database [31]. IS elements are relatively short genetically compact DNA segments (between 0.7 and 2.5 kb) encoding no functions other than those involved in their mobility within the genome [32]. IS*Psyr31* is predicted to have two, partially overlapping reading frames associated with a −1 frame shift; this is the pattern found in IS*3*/IS*911* type elements [33]. Amplicon sequencing of selected sites in Pac_ICE1 confirmed that all 14 New Zealand strains apparently carry identical islands (with the exception of the IS detected in strain 6.1). The fully assembled Pac_ICE3 sequences of two Chilean strains (ICMP19439 and ICMP19455) are identical to each other. Amplicon sequencing of selected sites in the island confirmed that all five Chilean strains apparently carry identical islands. The island sequence of the Italian strains, ICMP18744, CRA-FRU8.43 and CFBP7286, are probably identical, but the assemblies in Genbank have several gaps. The Pac_ICE1 of the Chinese strain M7/CH2010-6 generated in the present study is apparently identical to the CH2010-6 sequence previously deposited in Genbank, but again the Genbank sequences of CH2010-6 are represented on several contigs. The genome sequence of CH2010-6 is reported to be identical to those of CH2010-5 and CH2010-7 [23]. Pac_ICE2 in the Chinese strain M228 (this study) is very different from Pac_ICE1 in other Chinese strains (M7/CH2010-6, CH2010-5 and CH2010-7), but is identical to Pac_ICE2 in the Italian strains such as ICMP18744.

The sequence of Pac_ICE1 from the Chinese strain M7/CH2010-6 is almost identical to that of the New Zealand PSA island sequences. The only difference between the M7 (China) Pac_ICE1 sequence and those in the New Zealand strains is the presence in the fully assembled M7 island of an IS*Psy31* element, beginning at position 63621. This IS*Psyr31* element is present within the ∼16 kB transposon, Tn6212 (Figure 5). In summary, the Pac_ICE sequences present an abundance of highly discriminatory SNPs (approximately 10,000 per island) and these SNPs allow the construction of a very robust PSA phylogeny.

#### Evidence of ICE mobility

Pac_ICE1 is integrated at a lysine tRNA coding gene adjacent to ClpB. The Pac_ICE1 is flanked by one full length lysine tRNA gene (*attL,* 76 bp) and one highly characteristic truncated lysine tRNA (*attR*, 51 bp). A second 76 bp lysine tRNA locus adjacent to exsB is not occupied by an ICE in M7 or the New Zealand strains. In contrast Pac_ICE2 and Pac_ICE3 are integrated at the lysine tRNA locus adjacent to exsB (they are flanked by a full length lysine tRNA gene and a 51 bp truncated lysine tRNA locus). The Italian, Chilean and M228 Chinese strains have a second unoccupied full length lysine tRNA coding gene adjacent to ClpB. There are only two lysine tRNA coding genes in these PSA sequence assemblies.

**Figure 6:**
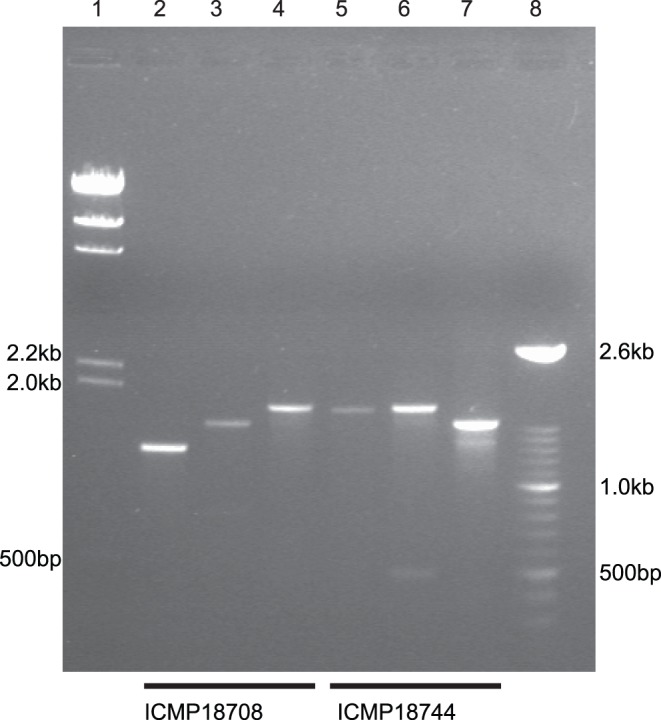
Evidence of Pac_ICE excision from the PSA genome. PCR was carried out with genomic DNA from ICMP18708 (lanes 2–4) or from ICMP18744 (lanes 5–7). Lane 1: molecular weight markers. Lanes 2 and 5: amplicons generated with internal ICE primers facing out into the genomic context. Lanes 3 and 6: amplicons generated using primers in the genomic context flanking each Pac_ICE. Lanes 4: amplicon generated using primers flanking the lysine tRNA gene near exsB (unoccupied in ICMP18708). Lane 7: amplicon generated using primers flanking the lysine tRNA gene near ClpB (unoccupied in ICMP18744). Lane 8∶100 bp molecular weight ladder.

If the ICEs described here are still capable of mobilization, two predictions can be made. Firstly, there should be some bacteria in which the ICE has excised from the genome, converting the occupied tRNA lysine site to an ‘empty’ site. Secondly, there should be some free, circular elements where a single *attR* site completes the circle. To explore these predictions, PCR was performed using the New Zealand PSA strain ICMP18708 and the Italian strain ICMP18744, with appropriate primers to detect the excised forms and the ‘empty’ target sites (Figure 6). The resulting amplicons were sequenced; this confirmed the presence both of empty target sites and free, circular, ICEs with an *attR* site (Figure S5). This molecular evidence consolidates the belief that the elements are active integrative conjugative elements.

#### Encoded ICE functions

The majority of the ORFs in the ICEs are predicted to be involved in ICE functions such as a XerC/D tyrosine recombinase (which lies adjacent to the tRNA lysine attachment site), pilus formation and DNA processing; these functions form what has been termed the ‘backbone’ of ICEs [34]. However, there are ORFs that are predicted to affect the phenotype of the pathogen. For example, Pac_ICE1 of the New Zealand isolates and M7 has a distinctive ORF encoding a C5-specific DNA methylase, while Pac_ICE2 in M228 and the Italian strains encodes at least three arsenate resistance proteins. Possibly reflecting the presence of these genes, the Italian strain, ICMP18744, grew on King’s B medium with 5 mM sodium arsenate, while the growth of the New Zealand strain was inhibited at 0.5 mM arsenate. In addition to the ORFs of known or predicted function, there are numerous hypothetical ORFs that are conserved in other *Pseudomonas* species and some ORFs of unknown function that have no counterparts in any *Pseudomonas*. For example Pac_ICE2 includes a large (6 kb) ORF that bears no close resemblance to any annotated gene.

The Tn6212 transposon occupies the same position (adjacent to a DNA topoisomerase III gene) in all of the ICEs (Figures 4 and 5). The first ORF in this transposon encodes a XerC-type tyrosine recombinase and the transposon has a 14 bp direct repeat (AAATACGTTATCAC) at its boundaries in all of the Pac_ICEs. Tn6212 is apparently an autonomously mobile XerC transposon that has very recently spread among the Pac_ICEs. This transposon, shared by Pac_ICE1, Pac_ICE2 and Pac_ICE3, encodes numerous ORFs likely to influence phenotype; encoding for example, a C4 dicarboxylate transporter, inorganic pyrophophatase, enolase, a voltage gated chloride channel, mercury resistance and a methyl accepting chemotaxis protein. The conserved position of the XerC transposon presumably reflects a specific integration site preference. The other mobile elements located within the ICE are inserted at various positions that may reflect a less specific integration site preference. Tn6211 is located in Pac_ICE1, Pac_ICE2 and Pac_ICE3 at one of three distinct sites (Figure 5); this is apparently the only copy of this element in the PSA genome. IS*Psyr31* is found in the Pac_ICE1 element of one New Zealand strain, 6-1, and at a different site in the Pac_ICE1 element of the Chinese strain M7 (Figure 5). This IS element is present in multiple copies in the core genomes of the New Zealand, Italian, Chilean and Chinese Psa.

#### ICEs in other *Pseudomonas syringae*

The highly conserved sequence of the core genome and the divergent sequence of the ICEs suggest that the ICEs have been very recently acquired by horizontal transfer from separate donor strains. To explore this possibility, we determined if ICEs were present in the Japanese strains of PSA and other members of the *P. syringae* species complex. Sequences indicating the presence of a putative ICE were found in PA459, but there was no convincing evidence of this island type in any other PSA strain from Japan. The ICE present in PSA PA459 (Pac_ICE4) has the characteristic lysine tRNA flanks (76 bp and 51 bp) and some of the ORFs found in other Pac_ICEs. It is integrated at the same lysine tRNA as Pac_ICE1. However Pac_ICE4 lacks the large transposon, Tn6212, common to all of the other Pac_ICEs.

The sequences of *Pseudomonas syringae* in Genbank were screened for similar ICEs. Some of the strains are fully assembled, while others are represented by unordered contigs. Putative ICEs carrying similar ORFs and *att* sites were detected in several pathovars of *P. syringae* (Table 7). The occurrence of these ICEs was sporadic and the sequence similarity of the ICEs did not correlate with the phylogeny of the host *Pseudomonas syringae*. For example we analysed the sequences of *P. syringae* pv. *avellanae* strains present in Genbank [35]. An ICE resembling Pac_ICE1 was present in the sequence of *P. syringae* pv. *avellanae* Ve013 (Figure 5) but not in *P. syringae* pv. *avellanae* Ve037 (a strain also in phylogroup 2) or the distantly related phylogroup 1 strain, *P. syringae* pv. *avellanae* BP631. The ICE in *P. syringae* pv. *avellanae* Ve013 (phylogroup 2) is very similar to the Pac_ICE1 sequence present in the New Zealand PSA (phylogroup 1). The ICE from *P. syringae* pv. *avellanae* Ve013 has a transposon (Tn6213) that is similar to Tn6211 (4837/4875). Tn6213 is located at a unique position (Figure 5) and, in consequence, has a different 6 base-pair target site duplication. The ICE from *P. syringae* pv. *avellanae* Ve013 has a Tn6212 transposon differing by one SNP from Tn6212 in the Pac_ICEs. The Tn6212 in the ICE from *P. syringae* pv. *avellanae* Ve013 is at the same position as Tn6212 in the Pac_ICEs. Apart from the SNPs present in these transposons, the ICE in *P. syringae* pv. *avellanae* Ve013 differs from Pac_ICE1 by only 12 SNPs, 3 small indels and a 22 bp repeat element variant.

**Table 7:** Sources of the seven ORF sequences from the ICEs of the *Pseudomonas syringae* pathovars used in the phylogenetic analyses.

ICE name	Strain	Source contigs
Pac_ICE1	CH2010-6	AGUH01000362, 363, 365, 096
Pac_ICE2	CRA-FRU8.43	AFTG01000003, 009
Pac_ICE2	CFPB7286	AGNO01000422, 423
Pac_ICE4	PA459	AGNQ01000546, 547, 394, 396
PsCit7_ICE	*P. syringae* Cit7	AEAJ01000143, 481, 490
Psyr GI-6	*P. syringae* pv. *syringae*	NC_007005∶1604346–1724178
Pspanici_ICE	*P. syringae* pv. *panici*	ALAC01000019, 062
Psoryzae_ICE	*P. syringae* pv. *oryzae*	ABZR01000735, 737, 738, 750
Psavellanae_ICE	*P. syringae avellanae* Ve013	AKJC01000059, 061, 063
Psjaponica_ICE	*P. syringae* pv. *japonica*	AEAH01001329, 1417, 1016, 1018, 1020, 1029, 1766
Pstheae_ICE	*P. syringae* pv. *theae*	AGNN01001232, AGNN01000825
Psgly_ICE	*P. syringae* pv. *glycinea* B076	AEGG01000011, 12
Psaesculi_ICE	*P. syringae* pv. *aesculi* NCPPB3681	ACXS01000730, 729, 728, 167

In contrast, comparison of the Pac_ICE of PSA with the ICE detected in the very closely related bacterium *P. syringae* pv *theae* indicated that the two ICE sequences were very divergent. The sequences were only approximately 85% identical and the *P. syringae* pv. *theae* ICE lacked the transposons Tn 6211 and Tn6212. However, most related ICEs carried transposons very similar Tn6212. For example, PsyGI-6, the ICE in *P. syringae* pv. *syringae* B728a, has a Tn6212 transposon with a 16312/16314 match to the PSA Tn6212 (Figure 4). *P. syringae* pv. *panici* has a Tn6212 transposon (16309/16316 match to Tn6212 in PSA). This suggests that this XerC transposon has recently spread, not only among Pac_ICEs, but more widely among other *Pseudomonas syringae* ICEs, integrating at a homologous sequence in the ICEs.

**Figure 7:**
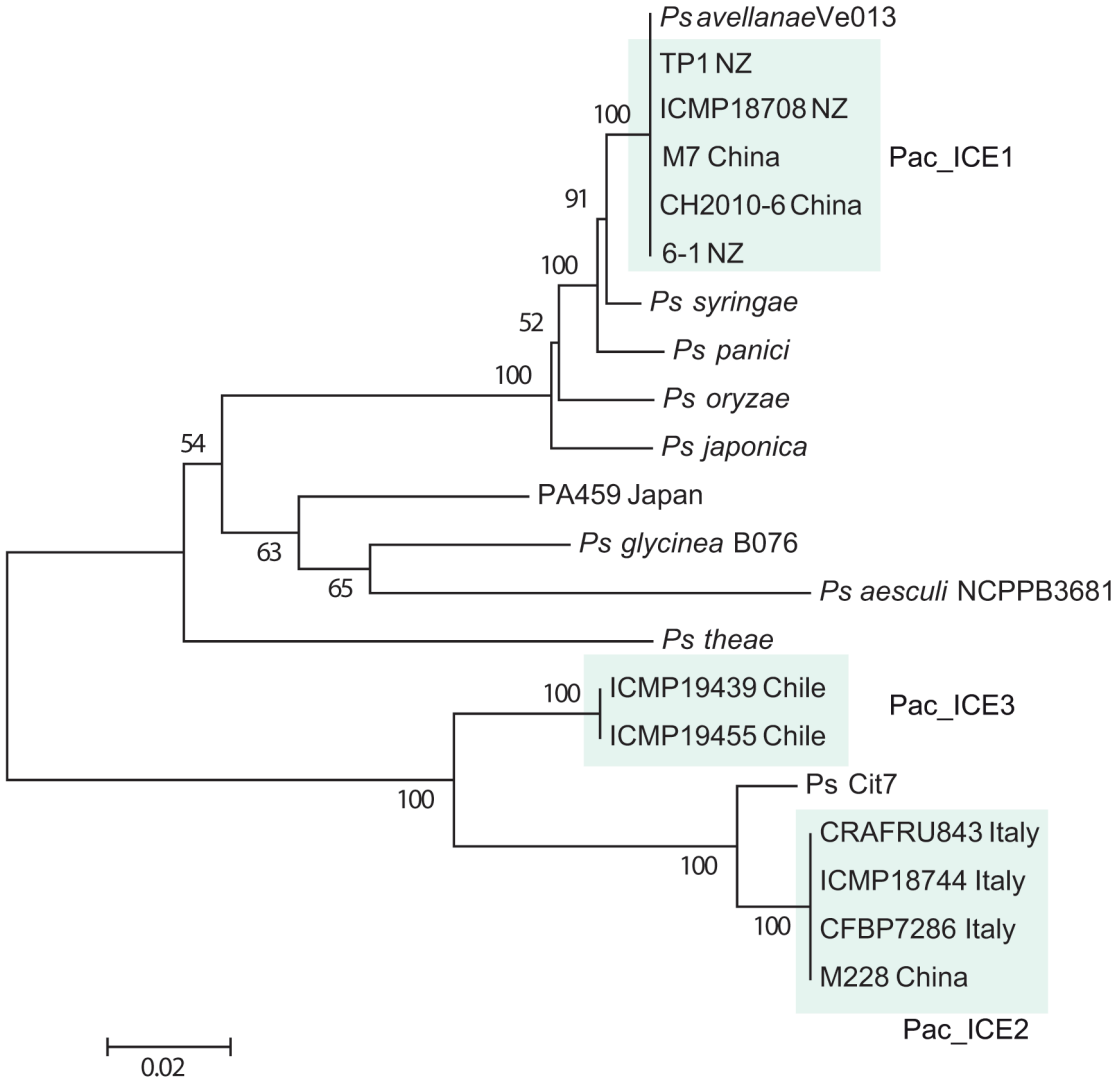
Evolutionary relationships of ICEs within strains of the *P. syringae* complex. The concatenated sequences of seven proteins (XerC/D tyrosine recombinase, ICE_TraI, DSBA oxoreductase, uvrD helicase, AdoMet-methyl transferase, DnaB and ParA (chromosome partitioning) encoded in the ICEs were used to generate the phylogenetic tree. The data for islands in strains other than those sequenced here were obtained from the accession numbers shown in Table 7. The evolutionary history was inferred using the Neighbour-Joining method in MEGA5 [26]. The optimal tree with the sum of branch length = 0.542 is shown. The percentage of replicate trees in which the associated taxa clustered together in the bootstrap test (1050 replicates) is shown next to the branches.

Seven ORFs represented in all these ICEs were selected for a phylogenetic analysis (Table 7 and Table S3). The protein sequences encoded by these ORF sequences were concatenated and the phylogenetic relationships shown in Figure 7 were generated. To avoid the possibility that different ORFs might have different phylogenetic histories, the relationships were also determined for each individual ORF (Figure S6). The concatenated data and that from the individual ORFs provide trees of similar topographies. These analyses indicate that the Pac_ICE1 is very similar to the ICE in *P. syringae* pv. *avellanae* Ve013 and *P. syringae* pv. *syringae* strain B728a. This suggests that these ICEs share a recent common molecular ancestor. In striking contrast, the Pac_ICE2 element in M228 and the Italian PSA strains is closely related to the ICE from *P. syringae* Cit7. The Pac_ICE3 present in the Chilean strains is not closely similar to any of the described *Pseudomonas syringae* ICEs. Pac_ICE4 present in strain PA459 is most similar to the ICE in *P. syringae* pv. *glycinea* B076.

## Discussion

High throughput sequencing of PSA genomes enabled the generation of a phylogenetic tree based on the alignment of 1,186 core genome protein sequences [23]. This placed PSA in a precise phylogenetic context within the *P. syringae* species complex. These authors then used SNP analyses to distinguish four groups of PSA isolates; from Japan, from Korea, from Europe and from China. The analysis of these sequences left unresolved the relationship between both the New Zealand and the Chilean PSA strains and other isolates. In order to obtain further insight into the origin of the current New Zealand kiwifruit bacterial canker epidemic, we used Illumina sequencing of multiple PSA genomes from four different countries. Using multiplex sequencing of eight P. syringae genomes per flowcell channel on an Illumina Hi-Seq it is possible to obtain draft genomes of ∼300x read depth that are of sufficient quality for SNP analysis.

Whole-genome analysis of these and other PSA sequence data present in the public databases indicates that the strains are closely related. Phylogenetic analysis of four loci from the PSA core genomes indicates that the PSA strains cluster in a monophyletic clade, with *P. syringae* pv. *theae* as the most closely related outgroup strain (as described by Mazzaglia et al. [23]). Japanese PSA strains are clearly distinct from other strains of PSA in this analysis, although PSA isolated from an earlier (1992) outbreak in Italy are members of the Japanese lineage [2], [23]. Other strains of *P. syringae* isolated from kiwifruit that are not associated with bacterial canker (PsD and PsHa) are more distantly related. We refer to these as *P. syringae* strains as PsD and PsHa to clearly differentiate both groups from the bacterial canker PSA strains. Neither PsD nor PsHa strains carry ICEs similar to those present in all of the PSA strains.

The presence of shared SNP sequences in the core genomes suggests that three distinct groups of PSA can be recognised; the New Zealand group, the Italian group and the Chilean group. The members of each of these groups carry multiple distinctive SNPs present in all members of the group and not found in strains outside of the group. The Chinese strains in our analysis do not carry these distinctive (geographically restricted) SNPs, nor are the SNPs present in the Japanese strains. The close similarity of these genome sequences indicate a very recent common ancestor for the Italian, Chilean and New Zealand lineages and the very similar strains that exist in China. By comparing the SNPs and using the Japanese sequences as an out-group, it is possible to determine the original and the derived sequence for each SNP. This indicates that the New Zealand, Italian and Chilean strains all have derived, distinctive SNPs. For example, all fourteen of the New Zealand strains have a characteristic 22 bp deletion that is not found in any other PSA and it must, therefore, be a derived characteristic. It follows that the recent outbreak in New Zealand is due to a distinct clone, as are the outbreaks in Italy and Chile. These outbreaks are not derived from each other. Furthermore, while the New Zealand outbreak can be derived from a strain similar to M7 from China, the reverse hypothesis is untenable since M7 does not carry the SNPs characteristic of and unique to the New Zealand strains (for example, the 22 bp deletion). Similarly, the hypothesis that the Chinese strains are derived from Italy is untenable since the Chinese strains do not carry the SNPs characteristic of the Italian PSA strains. The diversity of strains in China in regard to SNPs and to the ICEs reveals higher diversity of PSA compared to NZ or Europe or Chile, clearly suggesting that PSA originated in China. These data strongly support a model whereby the Italian, New Zealand and Chilean outbreaks each had a separate clonal origin from a diverse Chinese PSA population. This is consistent with a previous proposal that PSA was recently transferred from China to Europe [23].

It is of interest to estimate the time since these PSA strains shared a common ancestor. This estimation can be achieved by using data from previous similar outbreaks of bacterial disease. For example, an outbreak of bleeding horse-chestnut canker was first noticed in Europe in 2002/3 and three strains of *P. syringae* pv. *aesculi* isolated from infected trees in the UK between 2006 and 2008 have been analysed ([36], referred to by [24]) This analysis, comparing three strains, identified 3 SNPs in approximately 2.7 Mb. Assuming the outbreak had a clonal point source, the time between the last common ancestor and isolation of the strains must be between 2 and 5 years. Based on these data, the three *P. syringae* pv. *aesculi* strains appear to have accumulated somewhere between 0.15 and 0.35 SNP per Mb per year. It is reasonable to assume similar population dynamics and a similar mutation rate for PSA as for *P. syringae* pv. *aesculi*, since they are closely related pathogens of woody plants producing similar pathology. Using the *P. syringae* pv. *aesculi* mutation rate estimate of 0.15–0.35 SNP/Mb/year, the time since the last common ancestor and time since the foundation of the specific outbreaks of PSA can be approximated. If 4.5 Mb of the genome sequences of two PSA strains from New Zealand (ICMP18708 and 6.1) are compared, three idiosyncratic SNPs are apparent, indicating that these strains had a common ancestor 2–4 years ago. Comparison of other New Zealand strains yields a similar degree of divergence. If 4.5 Mb of the genome of an Italian strain (ICMP18744) are compared to ICMP18708 (NZ) twelve differences are apparent, approximately 2.5 SNPs per Mb. These comprise the geographically distinctive SNPs (8) and the idiosyncratic SNPs (4). This suggests the Italian and New Zealand strains had a common ancestor about 10–15 years ago. These estimates are obviously imprecise; the important point is that all comparisons between the core genomes of PSA from China, New Zealand, Chile and the recent isolates from Italy indicate that these strains shared a recent common ancestor and that the outbreaks had an even more recent clonal origin. Mazzaglia et al. [23] found 7000 SNPs between the Japanese/Korean and the late Italian/Chinese lineages. This suggests these lineages diverged at a much earlier time, approximately 10,000 years ago.

While the core genomes of the PSA isolates from New Zealand, Chile, China and Italy (the recent outbreak) were almost identical, the ICEs contained an abundance of genetic variation when strains from different geographical origins were compared. These ICEs fall into four types; Pac_ICE1 is present in all of the New Zealand PSA strains, the Chinese strain M7/CH2010-6 and the Chinese strains sequenced previously (CH2010-5, CH2010-7) [23]. Pac_ICE2 is present in the Italian strain ICMP18744, and in two Italian strains sequenced previously [2], [23]. An identical Pac_ICE2 element is present in M228, the second Chinese isolate sequenced in the present study. Pac_ICE3 is present in the five PSA strains from Chile. A fourth element, Pac_ICE4 is found in PA459, a PSA strain from Japan. The abundant genetic variation among Pac_ICE1, Pac_ICE2 and Pac_ICE3 permits the creation of a robust phylogeny that supports the groupings found using the SNPs in the core genomes of PSA. The Pac_ICE analyses also permit the grouping of the New Zealand PSA with the Chinese strain M7, CH2010-5 and CH2010-7. This clear recognition of the close relationship between the Chinese strains such as M7 and the New Zealand PSA was not possible using the very rare SNPs in the core genomes. The different ICEs typically carry 10,000 different SNPs in their ∼100 kb sequences, of which only about 70 kb can be aligned. Comparison of Italian and New Zealand PSA strains indicates that the core genomes have about 0.25 differences per 100 kb. The ICEs are, therefore, ∼50,000 times as divergent as the core genomes. Many of the substitutions are neutral, third codon, substitutions and this process of substitution must be approaching equilibrium. It follows that the extrapolated time to the most recent common ancestor of the ICEs must be >50,000 times as great as the time since the core genomes shared a common ancestor. It is remarkable that such divergent elements should have been taken up by horizontal transmission into the PSA genome on three separate recent occasions. Identical Pac_ICE2 elements are found in the Italian clone and in the rather dissimilar Chinese strain, M228. The Italian strains and M228 differ at ∼1 position in 16 kb, but their ICEs are identical across ∼108 kb. This suggests that Pac_ICE2 (and by implication, the other Pac_ICEs) has been transferred from one PSA strain to another.

It is interesting to consider the possible sources of the ICEs in PSA. Pac_ICE1 is very similar to the ICE present in *Pseudomonas syringae* pv. *avellanae* Ve013 (Figure 7). Bacterial canker disease affecting hazelnut is a newly emerged disease, first described in Greece and Italy in the mid 1970s [35]. There are at least two, quite distinct, types of *P*. *syringae* pv. *avellanae*, one represented by strains Ve013 and Ve037 and the other type by strains such as BP631. Although an ICE is present in strain Ve013, there is no ICE of this type in strain Ve037 or BP631. The close similarity of Pac_ICE1 and the ICE in *P*. *syringae* pv. *avellanae* Ve013 indicates that these elements recently had a common origin. The unique position and sequence of the Tn6211 transposon in the Pac_ICE1 compared with the related transposon Tn6213 in *P. syringae* pv. *avellanae* indicates that Pac_ICE1 cannot be derived from the ICE in *P. syringae* pv. *avellanae* Ve013, nor can it have given rise to this *P. syringae pv. avellanae* ICE.

The Pac_ICE2 element in the Italian PSA strains and M228 from China is similar to the ICE from *P. syringae* Cit7 (phylogroup 2) which suggests that Pac_ICE2 may be derived from a strain closely related to this *P. syringae* Cit7. The Pac_ICE3 present in the Chilean strains is not closely similar to any of the described *P. syringae* ICEs. Pac_ICE4 from strain PA459 is closest to the ICE in *P. syringae* pv. *glycinea* B076. Collectively the data indicate multiple transfers of ICEs between distantly related *P. syringae* pathovars.

This description of the phylogenetic relationships of the ICEs is further complicated by the presence within the divergent ICEs (Pac_ICE1, 2 and 3) of a highly conserved XerC transposon, Tn6212. Similar transposon sequences are also present in the ICEs of other *P. syringae* pathovars. The close similarity of these Tn6212 sequences in various divergent *P. syringae* pathovars indicates that this XerC transposon has recently been transmitted between different ICEs, integrating at a specific site recognised by the XerC-type tyrosine recombinase found within the transposon. The Tn6211 is also conserved among the ICEs, suggesting it may also be a recent acquisition by the ICEs.

Taken together with the core genome analysis (which suggested a very recent common ancestor for the PSA) the analysis of these ICEs suggests the following hypothesis. The PSA strains from Italy, New Zealand, Chile and China share a common ancestor in existence no more than a few decades ago. During this time three related but distinct ICEs were acquired through horizontal transfer. It is important to stress that these ICEs are not simply examples of selfish DNA; they carry ORFs whose products will modify the phenotype of the PSA. The acquisition of these elements may, therefore, reflect not only the dynamic nature of a transmissible selfish element but also selective advantage conveyed by such an element. It is possible that the common PSA ancestor, a strain lacking any ICE, may still exist in China. Isolating and characterising such a strain would clarify the phenotypic impact of the ICEs.

Our study contributes to the description of the emergence and spread of PSA. Specifically, analysis of the substitutions present in the core genomes and analysis of the abundant variation present in the integrative conjugative elements (ICEs) provide evidence that the source of the New Zealand, Italian and Chilean outbreaks is China, supporting and extending the hypothesis of Mazzaglia et al. [23]. This conclusion is based on two, independent lines of evidence: analysis of the substitutions present in the core genomes and analysis of the abundant variation present in the integrative conjugative elements (ICEs). Three extensions of this work are obvious. Firstly it is important to describe the diversity of PSA present in China. It is of interest to discover whether there are only a few or many PSA types in China, possibly carrying a wider diversity of ICEs. Secondly, the very recent invasion of the PSA genome by these ICEs requires some explanation. One possibility is that the acquisition of an ICE (on three separate occasions) triggered the emergence of an aggressive pathogen. Thirdly, it is important to determine if the different ICEs are conferring distinct phenotypes on the PSA strains, which might for example contribute to pathogenicity. One consequence might be that strains of kiwifruit resistant to one type of PSA might not be resistant to another. Additionally, some PSA may be inherently more virulent as a result of the particular ICE they carry. Although there is no significant diversity in the core genome, the diversity in the ICE regions suggests that strict border control by kiwifruit producing countries remains imperative. Cai et al., [37] have recently advocated the importance of border control in restricting the movement of *P. syringae* pv. *tomato* strains and other plant pathogens.

## Materials and Methods

### Bacterial Strains, growth, and DNA Extraction

Bacterial strains are listed in Table 1. Two Chinese strains in the present analysis are of particular significance and their origin is as follows: both were isolated from *Actinidia chinensis* var. *rufopulpa* Hong Yang (the red-centred kiwifruit) in Wei County, Shaanxi province. M7 was isolated from leaves in June 2010, while M228 was isolated from a branch in December 2010 (Zhibo Zhao, pers. comm). The Chilean strains represent diverse isolations; for example ICMP19456 was isolated from *Actinidia chinensis*, while the other four PSA strains are from different varieties of *A. deliciosa*. Previously unpublished strains of PSA from New Zealand were isolated from infected kiwifruit and confirmed as PSA by their weak fluorescence on King’s B medium and by PCR of the 16S genome region. PsD strains are strongly fluorescent on King’s B medium, whereas PSA and PsHa are only weakly fluorescent. The designation was confirmed by amplicon sequencing of the *gltA* gene. PSA, PsD and PsHa strains have characteristic *gltA* sequences. For DNA isolation, bacteria were grown on King’s B agar for 72 hours at 26°C. DNA was isolated using the Mo Bio™ Microbial DNA Isolation Kit (GeneWorks NZ, Ltd) following the manufacturer’s instructions.

### Genome Sequencing

Sequencing libraries were prepared using the Illumina TruSeq DNA Sample Preparation v2 kit following the standard low throughput protocols. Briefly, 1 ug of each genomic DNA was sheared to approximately 350 bp size using the Covaris S2 (Covaris, Woburn, MA, USA) with the following conditions: Intensity 4; Duty cycle 10%; 200 bursts per cycle; and 120 s total time. End repair, A-tailing and addition of barcoded adaptors were performed as per the standard low throughput protocol. Ligation products were run on a 2% 1× TAE gel and bands of 400–500 bp were excised, purified and enriched by PCR amplification following Illumina’s protocols. The final libraries were quantitated using the Bioanalyzer 2100 DNA 1000 chip (Agilent, Santa Clara, CA. USA) and the Qubit Fluorometer using the dsDNABR kit (Life Technologies, Burlington, ON. Canada).

PE sequencing was performed on various Illumina platforms: New Zealand, Italian and Chinese strains were sequenced to 100 bp on a HiSeq 2000 system and demultiplexed using the Illumina Casava application, v 1.8.2. The two strains from Chile were sequenced to 150 bp reads on a MiSeq system.

### *De novo* Assembly

The Edena assembler v110920 [38] was run with default settings on a RedHat Linux server, using the “paired” setting to allow read pairing to be taken into account. For assemblies generated using 150 bp paired end reads, an internal application limit required that reads were trimmed to 128 bp, however this was not required for strains sequenced using 100 bp paired-end reads.

### Genome-wide Comparisons of PSA

SeqMonk was used to provide an overall comparison of the newly sequenced PSA genomes. SeqMonk is a program that enables the visualisation and analysis of mapped next generation sequence data (http://www.bioinformatics.babraham.ac.uk/projects/seqmonk/).

### Genome Rearrangement Comparisons

Genomic islands were compared on a pairwise basis using the Artemis Comparison Tool, release 11.0.0, [27] run on a Mac Pro under MacOS X 10.7 after pairwise BlastN comparisons performed by creating a Blast database of each sequence and running *blastall –p blastn –m 8* to generate a tabbed list of HSPs (high-scoring segment pairs). Further visualisation was performed with the Mauve application, v 2.3.1, [28] run on a Mac Pro under MacOS X 10.7.

### Genome-wide SNP Detection

Comparisons were made between genomes using the bl2seq server at NCBI using the megablast setting. The total genome assembly of one strain was entered as the subject sequence, while each contig of the second strain was entered in turn as the query sequence. This continued until more than 4.5 Mb of the query genome sequence had been compared to the subject genome. We assessed the number of sequence differences between the strains by adding the observed SNPs and counting the insertion/deletion events as one difference. Only SNPs appearing in contigs reflected by coverage levels >100x were included in the analysis. Our method will underestimate the sequence differences between strains because any SNPs occurring near the ends of contigs will be ignored. However, the estimate is likely to be at least half of the actual SNPs.

A similar method was used to compare the sequences of the two PSA strains isolated in Italy in 2008 (CFPB7286 and CRA.FRU 8.43). Each contig from CFPB7286 (accession AGNO01000000) was used to query the assembled genome of CRA.FRU 8.43 (accession AFTG01000000) at the *Pseudomonas syringae* genome BLAST site. In this case, sequence differences in the terminal 60 bp of each contig were not included in the analysis; many of the contigs end in repeated sequences and it is obvious that there are assembly problems with the termini.

### SNP Analysis by PCR and Sanger Sequencing

Primers were designed manually based on the genome sequences available and are listed in Table S4. PCR reactions were done in 25 µL using a premixed ready-to-use solution containing a modified Taq DNA polymerase, dNTPs, MgCl2 and reaction buffers at optimal concentrations (ReddyMix, ThermoFisher), upstream and downstream primers at a final 1 µM concentration each, and about 40 ng of DNA template. The temperature profile for amplification consisted in an initial denaturation step at 94°C for 5 min, followed by 35 cycles at 94°C for 30 s, 50°C for 30 s and 72°C for 30 s, and by a last elongation step for 7 min. PCR products were visualised on 1.5% agarose gels under UV light and purified using QIAgen spin columns. The products were sequenced on both strands using the same primers used for amplification by Genetic Analysis Service (GAS; http://gas.otago.ac.nz) at the University of Otago, Dunedin, New Zealand. Any ambiguities were resolved through close inspection of the corresponding chromatogram (4Peaks; http://www.mekentosj.com/science/4peaks).

### Detection of Integrative Conjugative Elements (ICE) in PSA

The sequence of the *Pseudomonas syringae* pv. *phaseolicola* ICE (PPHGI-1; accession AJ870974) and that of the ICE PsyrGI-6 from *Pseudomonas syringae* pv. *syringae* 728a [19] were used to search our fully sequenced PSA genomes (bl2seq). The limits of the PSA genomic islands were determined by reference to the flanking lysine tRNA and truncated lysine tRNA sequences. The RAST server (http://rast.nmpdr.org) [39] was used to annotate these ∼100 kb regions.

### Identification and Naming of Mobile Elements in Pac_ICEs

Several types of mobile genetic element were identified within the integrative conjugative elements in the PSA genomes. Their limits were defined by detecting the inverted repeat sequences at their termini and the flanking target-site duplications created upon their integration. Two transposon elements (Tn6211 and Tn6212) have been assigned transposon numbers by the Transposon Name Registry at: http://www.ucl.ac.uk/eastman/research/departments/microbial-diseases/tn [29]. An IS element present in two examples of Pac_ICE1 (strains M7 and 6-1) has been assigned the name IS*Psy31* by the IS Finder website (www-is.biotoul.fr/) [31].

### Phylogenetic Tree Construction

We used the DNA sequences of four housekeeping genes to construct phylogenetic trees which include strains of *Pseudomonas syringae* isolated from kiwifruit vines, but which are not apparently highly pathogenic. These genes, rpoD, encoding sigma factor 70; gyrB, encoding DNA gyrase B; gltA (also known as cts), encoding citrate synthase; and gapA, encoding glyceraldehyde-3-phosphate dehydrogenase are a subset of the seven used in the original *P. syringae* MLST paper [13]. These loci have been used in several phylogenetic studies of *Pseudomonas* strains [14], [23], [40], [41], [42], [43]. The full-length sequences of each of the genes were obtained from our own Illumina sequence data and from the WGS database using blast searches. The primer sequences used for DNA amplification and Sanger sequencing of these four loci in strains that were not fully sequenced are shown in Table S4.

Sequences from each locus were aligned by using CLUSTALW2 (http://www.ebi.ac.uk/Tools/msa/clustalw2/) and trimmed to their minimal shared length in Seaview [44]. Neighbour-joining and maximum-likelihood phylogenetic analyses were performed on the individual and concatenated datasets by using MEGA5 [26]. 1050 bootstrap replicates were performed.

Phylogenetic analyses of the ICE sequences were also conducted. Seven ORFs were selected from among those held in common by the putative Pac_ICEs under investigation (Table 7 and Table S3). These ORFs were present in the same gene order in all of the ICEs and were representative of gene modules often found in conjugative elements [21], [45]. We searched for the presence of other putative ICEs in species related to *Pseudomonas syringae* pv. *actinidiae*, both at the *Pseudomonas syringae* genomes BLAST website (http://www.ncbi.nlm.nih.gov/genomes/geblast.cgi?taxid=317#SearchSet) and at the WGS. BLASTN searches suggested the presence of conjugative elements of this type in several *Pseudomonas syringae* strains (Table 7). Based on these searches, the contigs of each strain that matched Pac_ICE sequences were assembled into putative scaffolds. The ORFs encoded in these scaffolds were examined using ORF finder at NCBI (http://www.ncbi.nlm.nih.gov/gorf/gorf.html) and the orthologs of the seven ORFs used in phylogenetic analyses were determined. Phylogenetic analyses were performed using the individual gene sequences and with a concatenated dataset.

### Analysis of ICE Excision

Primer sequences were designed to amplify the regions flanking the two lysine tRNA genes in PSA (near ClpB and exsB). These were paired with each other (to detect *att* sites that were empty of Pac_ICEs) or with primers designed to anneal at sites within the ICEs but directed towards the flanking regions (positive controls). The primers annealing within the ICEs but directed towards the flanking regions were paired with each other to detect the presence of free ICE circles. Primers sequences are shown in Table S5.

### dS/dN Analyses

The DNA sequences of the representative seven ICE orfs from strains ICMP18708 (NZ) Pac_ICE1, ICMP18744 (Italy) Pac_ICE2, ICMP19455 (Chile) Pac_ICE3 were codon aligned and the number of synonymous and non-synonymous substitutions were calculated using the SNAP website (www.hiv.lanl.gov) [46]. Calculations were made using each ORF separately and with a concatenated sequence containing all seven ORF sequences.

## Supporting Information

Figure S1 **Overview of sequencing reads of PSA strains from this study.** Included are the four New Zealand PSA (TP1, 6.1, ICMP18800 and ICMP18708), one Italian PSA (ICMP18744) and the Japanese PSA (ICMP9853). The reads were mapped onto the assembled genome of *P. syringae* pv. *tomato* DC3000 (accession: NC_004578).
(EPS)

Figure S2 **PSA sequence reads which map to the 1–2 Mb region of** ***P. syringae*** **pv.** ***tomato*** **DC3000.** Those reads from the four fully sequenced New Zealand PSA (TP1, 6.1, ICMP18800 and ICMP18708), the one fully sequenced Italian PSA (ICMP18744) and the Japanese PSA (ICMP9853) which map onto the assembled genome of *P. syringae* pv. *tomato* DC3000 (accession: NC_004578) in the region from 1–2 Mb.
(EPS)

Figure S3 **Comparison of PPHG-1 from** ***P. syringae*** **pv.** ***phaseolicola*** **with PsyrGI-6 from** ***P. syringae*** **pv.** ***syringae*** **.** An Artemis Comparison Tool [27] alignment of the two island sequences: PPHGI-1 (accession: AJ870974) [20] and PsyrGI-6 [19]. The blue areas refer to regions that are inverted between islands.
(EPS)

Figure S4 **Dotplot comparisons of Pac_ICEs.** Dotplots were generated at the NCBI blast2 site (http://blast.ncbi.nlm.nih.gov/Blast.cgi?PAGE_TYPE=BlastSearch&PROG_DEF=blastn&BLAST_PROG_DEF=megaBlast&BLAST_SPEC=blast2seq). A. Comparison of Pac_ICE1 from ICMP18708 (NZ) with Pac_ICE3 from the Chilean strain ICMP19455. B. Comparison of the Pac_ICE1 sequence from ICMP18708 (NZ) with Pac_ICE2 from the Italian strain ICMP18744. C. A comparison of the Pac_ICE2 from ICMP18744 (Italy) with the Pac_ICE3 from the Chilean strain ICMP19455. The transposon Tn6211 is depicted as a green line, the Tn6212 transposon is coloured orange.
(EPS)

Figure S5 **Sequence data from free DNA circles derived from putatively excised ICEs.** PCR products were amplified using primers that annealed within the ICEs but were directed towards the flanking regions in order to detect the presence of free ICE circles. A. Sequence of the amplicon derived from excised versions/free, circular molecules of Pac_ICE1 from ICMP18708. B. Sequence of the amplicon derived from excised versions/free, circular molecules of Pac_ICE2 from ICMP18744. Blue: internal ICE sequence from the region near the XerC recombinase gene. Orange: internal ICE sequence from the region near the *ParA* gene. Shaded yellow: truncated tRNA lysine gene, *attR*.
(EPS)

Figure S6 **Phylogenetic trees constructed with the seven ORFs present in** ***P. syringae*** **ICEs.** These trees were generated separately for each ORF using the Neighbour-Joining method in MEGA5 [26]. The percentage of replicate trees in which associated taxa clustered together in the bootstrap test (1050 replicates) is shown next to the branches (only those values >55% are shown).
(EPS)

Table S1 **SNPs (single nucleotide polymorphisms) found among strains of PSA at loci used by Mazzaglia et al.** [**23**] **.**
(DOCX)

Table S2 **Single Nucleotide Polymorphisms (SNPs) present in only one of the New Zealand strains of PSA (idiosyncratic SNPs).**
(DOCX)

Table S3 **The names and positions of the seven ORFs in the Pac_ICEs used in the dS/dN analyses and in the phylogenetic analyses of the ICEs.**
(DOCX)

Table S4 **Primer sequences used to amplify regions of the core PSA genomes for SNP analyses and phylogenetic analyses.**
(DOCX)

Table S5 **Primer sequences used to analyse the Pac_ICE insertion sites and to detect the excised circular Pac_ICE molecules.**
(DOCX)
