# Supplementary material for: Pseudomonas syringae pv. actinidiae from Recent Outbreaks of Kiwifruit Bacterial Canker Belong to Different Clones That Originated in China
Source: PLoS One. 2013 Feb 27;8(2):e57464. doi: 10.1371/journal.pone.0057464 (PMC3583860; doi:10.1371/journal.pone.0057464)
Supplement: Table S1 — SNPs (single nucleotide polymorphisms) found among strains of PSA at loci used by Mazzaglia et al. [23] . (DOCX) [file pone.0057464.s007.docx]

**Table S1.** Single nucleotide polymorphisms (SNPs) in PSA at loci used by Mazzaglia et al. [23].

| Mazzaglia primer locus | | scaffold 176 | scaffold 237 | scaffold 398 | scaffold 452 | scaffold 911 | scaffold 190 |
| --- | --- | --- | --- | --- | --- | --- | --- |
| CH2010-6 | China | A | A | A | A | A | T |
| M7 | China | A | A | A | A | A | T |
| M228 | China | G | C | T | G | T | T |
| ICMP18800 | NZ | G | C | T | G | T | T |
| ICMP18708 | NZ | G | C | T | G | T | T |
| TP1 | NZ | G | C | T | G | T | T |
| 6.1 | NZ | G | C | T | G | T | T |
| ICMP18839 | NZ | G | C | T | G | T | T |
| ICMP18875 | NZ | G | C | T | G | T | T |
| ICMP18744 | Italy | G | C | T | G | T | C |
| CRAFRU8.43 | Italy | G | C | T | G | T | C |
| CFBP7286 | Italy | G | C | T | G | T | C |
| ICMP19439 | Chile | G | C | T | G | T | T |
| ICMP19455 | Chile | G | C | T | G | T | T |
| M302091 | Japan | G | C | T | G | T | T |
| ICMP9853 | Japan | G | C | T | G | T | T |
