# Supplementary material for: Pseudomonas syringae pv. actinidiae from Recent Outbreaks of Kiwifruit Bacterial Canker Belong to Different Clones That Originated in China
Source: PLoS One. 2013 Feb 27;8(2):e57464. doi: 10.1371/journal.pone.0057464 (PMC3583860; doi:10.1371/journal.pone.0057464)
Supplement: Table S2 — Single Nucleotide Polymorphisms (SNPs) present in only one of the New Zealand strains of PSA (idiosyncratic SNPs). (DOCX) [file pone.0057464.s008.docx]

**Table S2.** Single Nucleotide Polymorphisms (SNPs) present in only one of the New Zealand strains of PSA (idiosyncratic SNPs).

| NZ strain | 6.1 | ICMP18800 | ICMP18708 | TP1 |
| --- | --- | --- | --- | --- |
|  |  |  |  |  |
| SNP contig | 7 | 22 | 38 | 101* |
|  | 20 | 74* | 12 | 33 |
|  | 24 | 7* | 2 | 4 |
|  | 35 * | 88 | 58 | 50 |
|  | 38 | 94 | 35* | 69 |
|  | 45 | 37* | 3 | 44 |
|  | 56 | 73 | 55* | 21 |
|  | 77 | 81* | 20 | 16 |
|  | 80 | 106 | 49 | 39* |
|  | 149 | 115 | 117 | 170** |
|  |  |  |  |  |

The SNPs personal to a particular strain of PSA (that is, not present in any other strain) were found by comparing about 5Mb sequence from four New Zealand strains.

The asterisks indicate the strain with the derived, idiosyncratic, SNP (or SNPs).
