# Supplementary material for: Pseudomonas syringae pv. actinidiae from Recent Outbreaks of Kiwifruit Bacterial Canker Belong to Different Clones That Originated in China
Source: PLoS One. 2013 Feb 27;8(2):e57464. doi: 10.1371/journal.pone.0057464 (PMC3583860; doi:10.1371/journal.pone.0057464)
Supplement: Table S3 — The names and positions of the seven ORFs in the Pac_ICEs used in the dS/dN analyses and in the phylogenetic analyses of the ICEs. (DOCX) [file pone.0057464.s009.docx]

|  | Pac_ICE1-nz ICMP18708 NZ | Pac_ICE2-it ICMP18744 Italy | Pac_ICE3-cl ICMP19455 Chile |
| --- | --- | --- | --- |
| Accession | KC148184 | KC148186 | KC148188 |
| XerC/D tyrosine recombinase | 549-1952 | 443-1864 | 547-1962 |
| ICE_TraI | 1949-3865 | 1861-3786 | 1959-3899 |
| DSBA oxoreductase | 22774-23460 | 24808-25503 | 21266-21961 |
| uvrD_helicase | 33840-35297 | 34121-35584 | 30579-32042 |
| AdoMet-methyl transferase | 44171-45622 | 45980-47440 | 43833-45293 |
| DnaB | 97098-98444 | 105004-106353 | 96984-98333 |
| ParA (chromosome partitioning) | 99899-100759 | 107802-108662 | 99782-100642 |

**Table S3.** The names and positions of the seven ORFs in the Pac_ICEs used in the dS/dN analyses and in the phylogenetic analyses of the ICEs.
