# Supplementary material for: Pseudomonas syringae pv. actinidiae from Recent Outbreaks of Kiwifruit Bacterial Canker Belong to Different Clones That Originated in China
Source: PLoS One. 2013 Feb 27;8(2):e57464. doi: 10.1371/journal.pone.0057464 (PMC3583860; doi:10.1371/journal.pone.0057464)
Supplement: Table S4 — Primer sequences used to amplify regions of the core PSA genomes for SNP analyses and phylogenetic analyses. (DOCX) [file pone.0057464.s010.docx]

**Table S4.** Primer sequences used to amplify regions of the core PSA genomes for SNP analyses and phylogenetic analyses.

| SNP locus (in M7) | Primer (this study) | Primer sequence (5’-> 3’) |
| --- | --- | --- |
| c6 | 4-3679 | GCTTCATCGAGTGTTCACTCG |
|  | 4-4433 | cgaggtgaagacgaacaacg |
| c12 | 9-55713 | CTTGGTTGTATCTGGTGCACC |
|  | 9-56585 | cagatgcagcgtgattactgg |
| c40 | 23-53077 | CACTGACGGCAAGCTGAGC |
|  | 23-53998 | gctcaacaacctgtccctgc |
| c499 | 39-57716 | CGAATGTGCTGATCGGTACG |
|  | 39-58586 | acagcttgtgagcagattcctgc |
| c192 | 126-30959 | GTTTCAGGCACTGGAACAGC |
|  | 126-30366 | cgaagatggcgtgacaacctcg |
| c290 | 153-2227 | GTGCATGACGTGATCAACGAGC |
|  | 153-3091 | ccataacgctcacatcgacg |
| c195 | 195-30388 | CGCTATAGGGCTTGCACACC |
|  | 195-31086 | GAGAACTGGATATACGCATGG |
|  |  |  |
| *P.s theae* scaffold, contig, position | Mazzaglia primers^1^ |  |
| 176, 5, 6360 | 176_F | GGTCACCAGTACAACGCTCA |
|  | 176_R | ACCAGCCAATCCTTTACGTG |
| 237, 121, 1890 | 237_F | CTTGTCGTTCCATTCCATCC |
|  | 237_R | GGTATCGACAACGCCTCTTA |
| 398, 19, 3855 | 398_F | ACGAAGGCCTGTACCGAAGT |
|  | 398_R | CGACGGTCAGGAAGGTTATC |
| 452, 21, 2795 | 452_F | CCTGCGCTGACTGAAATCAT |
|  | 452_R | GACGTCATGACCTTGAGTTGTT |
| 911, 61, 832 | 911_F | GATAACCGCCCACCTGATAG |
|  | 911_R | ACGGCTATTACCCGCTCAAC |
| 190, 7, 4380 | 190_F | GTGACCGACTCGCTGAAAAG |
|  | 190_R | CGGATGTTCTACATGCGCTAC |
|  |  |  |
| Core Genome Loci |  |  |
| gapdh | GapA-F | AGTTGATCATCGAGGGCGCWGCC |
|  | GapA-Rv | CCCAYTCGTTGTCGTACCA |
| gltA | gltA-F | AGTTGATCATCGAGGGCGCWGCC |
|  | gltA-R | TGATCGGTTTGATCTCGCACGG |
| gyrB | GyrB-Fmib | GGYGGYAAGTTCGAYGACAAYTCC |
|  | GyrB-Rvmib | TRATYKCAGTCARACCTTCRCGSGC |
| rpoD | rpoD_F | AAGGCGARATCGAAATCGCCAAGCG |
|  | rpoD_Rv | GGAACWKGCGCAGGAGTCGGCACG |

^1^Primer sequences used to analyse SNPs found by Mazzaglia et al. [23] are taken from their Table 4.
