# Supplementary material for: Pseudomonas syringae pv. actinidiae from Recent Outbreaks of Kiwifruit Bacterial Canker Belong to Different Clones That Originated in China
Source: PLoS One. 2013 Feb 27;8(2):e57464. doi: 10.1371/journal.pone.0057464 (PMC3583860; doi:10.1371/journal.pone.0057464)
Supplement: Table S5 — Primer sequences used to analyse the Pac_ICE insertion sites and to detect the excised circular Pac_ICE molecules. (DOCX) [file pone.0057464.s011.docx]

**Table S5.** Primer sequences used to analyse the Pac_ICE insertion sites and to detect the excised circular Pac_ICE molecules.

|  |  | primer name |  | primer sequence (5’-> 3’) | |  |
| --- | --- | --- | --- | --- | --- | --- |
|  |  |  |  |  |  |  |
| ICMP18744 |  | Italy_30141 |  | GGTTACGCCTGCTACACCATGAGC | | |
|  |  | Italy_31675-r | | gtaactgaagcagaattgccagacg | | |
|  |  |  |  |  |  |  |
|  |  | Italy_138725 | | GGATTATCTGCAAGGCGCTTGG | | |
|  |  | Italy_140618-r | | cgatgctgccaatcgagagaatgc | | |
|  |  |  |  |  |  |  |
| ICMP18708 |  | NZ_32727 |  | GTATCACCGATATCCAGCTGG | | |
|  |  | NZ_34049-r |  | caggctgatcacttacgttgg | |  |
|  |  |  |  |  |  |  |
|  |  | NZ_133595 |  | GAACTGTGCTGTTGAGGACATCG | | |
|  |  | NZ_135130-r | | cagatctgtttggtgaactcgctgac | | |
